# High-Refractive-Index Materials for Giant Enhancement of the Transverse Magneto-Optical Kerr Effect

**DOI:** 10.3390/s20040952

**Published:** 2020-02-11

**Authors:** Edwin Moncada-Villa, J. Ricardo Mejía-Salazar

**Affiliations:** 1 Escuela de Física, Universidad Pedagógica y Tecnológica de Colombia, Avenida Central del Norte 39-115, Tunja 150003, Colombia; edwinmoncada83@gmail.com; 2 National Institute of Telecommunications (Inatel), Santa Rita do Sapucaí, MG 37540-000, Brazil

**Keywords:** HRI materials, magneto-optics, Fabry–Perot resonances

## Abstract

The ability of plasmonic structures to confine and enhance light at nanometer length scales has been traditionally exploited to boost the magneto-optical effects in magneto-plasmonic structures. These platforms allows for light control via externally applied magnetic fields, which is of prime importance for sensing, data storage, optical-isolation, and telecommunications applications. However, applications are hindered by the high-level of ohmic losses associated to metallic and ferromagnetic components. Here, we use a lossless all-dielectric platform for giant enhancement of the magneto-optical effects. Our structure consists of a high-refractive index dielectric film on top of a magnetic dielectric substrate. We numerically demonstrate an extraordinarily enhanced transverse magneto-optical Kerr effect due to the Fabry–Perot resonances supported by the high-refractive index slab. Potential applications for sensing and biosensing are also illustrated in this work.

## 1. Introduction

Owing to several potential applications in biosensing, optical isolation, mapping of microwave currents, and ultrafast optical data storage devices, magneto-optical (MO) effects have become a subject of considerable theoretical and experimental interest for many research groups [1,2,3,4,5,6,7,8,9,10,11,12,13,14,15,16,17,18,19,20,21,22,23]. In particular, the transverse MO Kerr effect (TMOKE) has emerged as a promising approach for improved biosensing [16,18], optical filtering [17], and magnetization monitoring [14,15]. In practice, many of these applications are challenged by extremely weak TMOKE signals in ferromagnetic metals (~$10^{-3}$) [24]. This limitation is traditionally beaten through the magnetoplasmonic effect, i.e., the combination of MO and plasmonic effects [1,2,3,4,5,6,7]. In this approach, the strongly enhanced near-field amplitudes of surface plasmon resonances (SPRs) are distributed inside an adjacent MO layer to improve the MO activity. However, this mechanism suffers from two important limitations. First, the need to use a prism coupler for SPR excitation hampers miniaturization and fabrication of portable plasmonic devices. The second drawback arises from large level of losses in noble and ferromagnetic metals at optical and infrared frequencies. Despite our earlier efforts on this topic [25,26], where we introduced the use of ε-near-zero materials, for giant enhancement of the TMOKE without the need to use prisms or grating couplers, applications are still challenged by optical losses associated with the presence of ferromagnetic metals.

**Figure 1:**
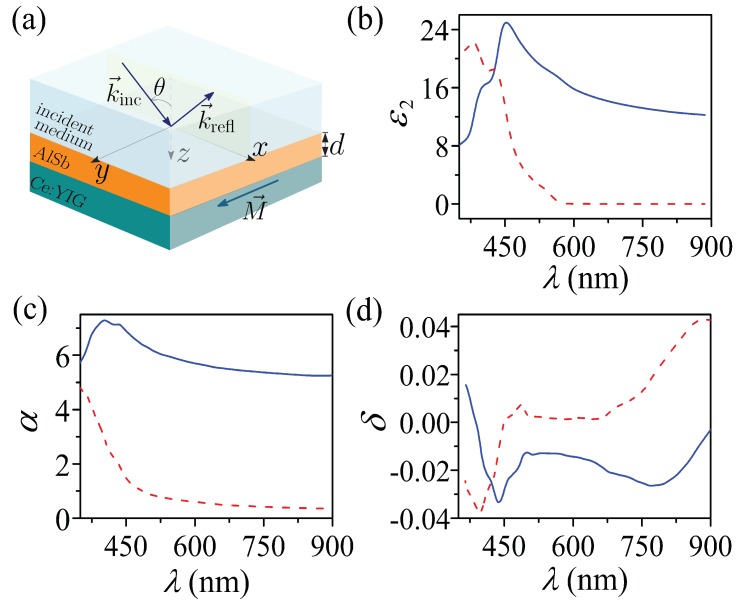
(color online) (**a**) Schematic of the dielectric system employed to enhance the transverse magneto-optical Kerr effect. The aluminium antimonide (AlSb) film is deposited on a magnetic Ce:YIG substrate. The magnetization M is considered along the *y*-axes, perpendicular to the incidence plane. (**b**) Real (solid) and imaginary (dashed) parts of the AlSb dielectric function ($\varepsilon_{2}$). The diagonal and off-diagonal elements of the Ce:YIG’s dielectric tensor (Equation (11)) are shown in panels (**c**,**d**). Real and imaginary components are represented by solid and dashed lines, respectively.

In this work, we present an entirely new concept for lossless TMOKE enhancement. Our strategy is as follows. We exploit the Fabry–Perot resonances, supported by a high-refractive-index (HRI) dielectric thin film, to provide giant TMOKE resonances (≈±1) using a dielectric MO substrate. The system under study is schematically represented in Figure 1a. The structure consists of a Ce:YIG (cerium-substituted yttrium iron garnet) MO substrate [27,28,29] covered by a film of high-refractive-index (HRI) material, AlSb in this case [30]. The incident medium is considered as air, with permittivity $\varepsilon_{1}=1.0$. In addition to the low-level of losses of the Ce:YIG, one of the major attractions is their fully integrability with III-V or Si-based semiconductors, which allows for on-chip MO applications [27,28,29].

TMOKE measures the relative change in the reflected light intensity when the structure is remagnetized, i.e., the magnetization is reversed from the positive (negative) to the negative (positive) sense, along the direction perpendicular to the plane of polarization [24]. This MO effect can only be observed for *p*-polarized (transversal magnetic) light, obliquely impinging over a system magnetized perpendicular to the incidence plane, and is calculated as

(1) $$\mathrm{TMOKE}=\frac{R_{pp}\left(m=+1\right)-R_{pp}\left(m=-1\right)}{R_{pp}\left(m=+1\right)+R_{pp}\left(m=-1\right)},$$

where $R_{pp}$ and m=+1 (m=−1) indicate the reflectance and the magnetization sense along the positive (negative) *y*-axis. Subindices pp are used to emphasize that polarization conversion (the presence of ps or sp terms) does not occurs in this MO configuration.

The reflectance and transmittance spectra for this structure are calculated, for *p*-polarized incident light, within the scattering-matrix method by [26,31]

(2) $$\begin{array}{ccc} R_{pp} & = & {\left|r_{pp}\right|}^{2}, \end{array}$$

(3) $$\begin{array}{ccc} T_{pp} & = & {\left|t_{pp}\sqrt{\frac{\beta_{1}}{\beta_{3}}}\right|}^{2}, \end{array}$$

where

(4) $$\begin{array}{ccc} r_{pp} & = & \frac{-\left(\left(\beta_{1}\beta_{2}-\beta_{2}\beta_{3}+\beta_{2}\gamma \right)\cos q_{2}d+i\left(\beta_{2}^{2}-\beta_{1}\beta_{3}+\beta_{1}\gamma \right)\sin q_{2}d\right)}{\left(\beta_{1}\beta_{2}+\beta_{2}\beta_{3}-\beta_{2}\gamma \right)\cos q_{2}d+i\left(-\beta_{2}^{2}-\beta_{1}\beta_{3}+\beta_{1}\gamma \right)\sin q_{2}d}, \end{array}$$

(5) $$\begin{array}{ccc} t_{pp} & = & \frac{2\beta_{2}\left(\beta_{3}-\gamma \right)}{\left(\beta_{1}\beta_{2}+\beta_{2}\beta_{3}-\beta_{2}\gamma \right)\cos q_{2}d+i\left(-\beta_{2}^{2}-\beta_{1}\beta_{3}+\beta_{1}\gamma \right)\sin q_{2}d}, \end{array}$$

with

(6) $$\begin{array}{ccc} \beta_{1} & = & \varepsilon_{1}^{-1/2}\cos\theta , \end{array}$$

(7) $$\begin{array}{ccc} \beta_{2} & = & \varepsilon_{2}^{-1}\sqrt{\varepsilon_{2}-\varepsilon_{1}\sin^{2}\theta }, \end{array}$$

(8) $$\begin{array}{ccc} \beta_{3} & = & \frac{\alpha }{\alpha^{2}-\delta^{2}}\sqrt{{\left(\frac{\alpha }{\alpha^{2}+\delta^{2}}\right)}^{-1}-\varepsilon_{1}\sin^{2}\theta }, \end{array}$$

(9) $$\begin{array}{ccc} \gamma & = & -\frac{m\delta }{\alpha^{2}-\delta^{2}}\varepsilon_{1}\sin\theta , \end{array}$$

(10) $$\begin{array}{ccc} q_{2} & = & \frac{\omega }{c}\sqrt{\varepsilon_{2}-\varepsilon_{1}\sin^{2}\theta }. \end{array}$$

The real and imaginary components of the permittivity for AlSb ($\varepsilon_{2}$) are presented with solid and dashed lines, respectively, in Figure 1b. The incidence plane is assumed along the xz-plane and the magnetization (**M**) parallel to the *y*-axis. Therefore, the permittivity tensor for the Ce:YIG MO material (${\hat{\epsilon }}_{3}$) can be written as

(11) $${\hat{\epsilon }}_{3}=\left(\begin{array}{ccc} \alpha & 0 & i\delta m\\ 0 & \alpha & 0\\ -i\delta m & 0 & \alpha \end{array}\right),$$

where m=+1 (m=−1) indicates the magnetization sense along the positive (negative) *y*-axis. The real and imaginary vales of α and δ are shown in Figure 1c,d by solid and dashed lines, respectively. From Figure 1, we can note a simultaneous lossless behavior, for the HRI film and the MO substrate, at working wavelengths (λ) larger than 600 nm. Therefore, we will use λ=795 nm, for which $\varepsilon_{2}=12.7928+0.0018679i$, α=5.3+0.4i and δ=−0.026+0.026i.

**Figure 2:**
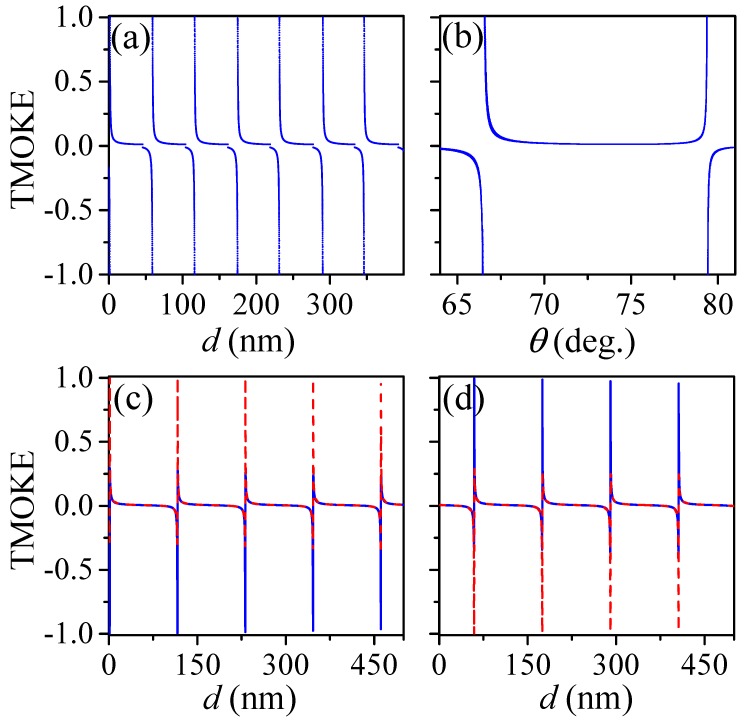
(color online) The highest possible TMOKE values are presented as a function of (**a**) the AlSb film thickness *d* and (**b**) the angle of incidence θ. Results are shown for (**c**) $\theta =66.45^{\circ }$ (solid line) and $\theta =66.57^{\circ }$ (dashed line), and (**d**) for $\theta =79.38^{\circ }$ (solid line) and $\theta =79.43^{\circ }$ (dashed line). The incident medium was considered as air ($\varepsilon_{1}=1.0$) in these calculations.

To investigate the TMOKE as function of the HRI film thickness (*d*) and the incident angle (θ), we calculated the highest TMOKE values for θ varying from $0^{\circ }$ to $90^{\circ }$, in steps of $\Delta \theta =0.01^{\circ }$, and for *d* from 0 nm to 400 nm, in steps of Δd=0.01 nm. For visualization purposes, results are presented in Figure 2a by a scatter plot as function of *d*. As noted from this figure, the first TMOKE resonances occur at very thin dielectric films of d=1.32 nm and d=1.51 nm, which are not considered here in order to avoid interface (AlSb/Si) effects due to the mismatch between the lattice parameters of Ce:YIG (~12.57 Å) and AlSb (~6.10 Å). In Figure 2b, on the other hand, results are replotted as function of θ. In contrast to periodic giant TMOKE signals (≈1) with increasing *d* (for $0^{\circ }\leq \theta \leq 90^{\circ }$), only four well-defined values of θ are observed in this latter figure (for 0≤d≤400 nm). Results for these θ-values are presented in Figure 2c,d as function of *d*. In particular, solid (dashed) lines in Figure 2c,d show the calculated TMOKE spectra, as function of *d*, for $\theta =66.45^{\circ }$ ($\theta =66.57^{\circ }$) and $\theta =79.38^{\circ }$ ($\theta =79.43^{\circ }$), respectively. An antisymmetric behavior is clearly observed for $\theta =66.45^{\circ }$ and $\theta =66.57^{\circ }$ ($\theta =79.38^{\circ }$ and $\theta =79.43^{\circ }$) in Figure 2c,d. Before we explain the mechanism behind these features, we must remember that electromagnetic waves propagating through the system in Figure 1a, with $\varepsilon_{2}>\varepsilon_{1}$ and $\varepsilon_{2}>\alpha$, undergoes multiple internal reflections inside the HRI film ($\varepsilon_{2}$). Constructive (destructive) interference of counter-propagating waves inside the HRI film produces a periodic set of maximum (minimum) transmission peaks ($T_{pp}\approx 1$), named Fabry–Perot resonances [32], with the period length described by [32]

(12) $$\Delta d=\frac{\lambda /2}{\sqrt{\varepsilon_{2}-\varepsilon_{1}\sin^{2}\theta }},$$

which, for simplicity, was calculated for a non-magnetized system (δ=0) in the symmetric configuration $\varepsilon_{1}≃\alpha$.

**Figure 3:**
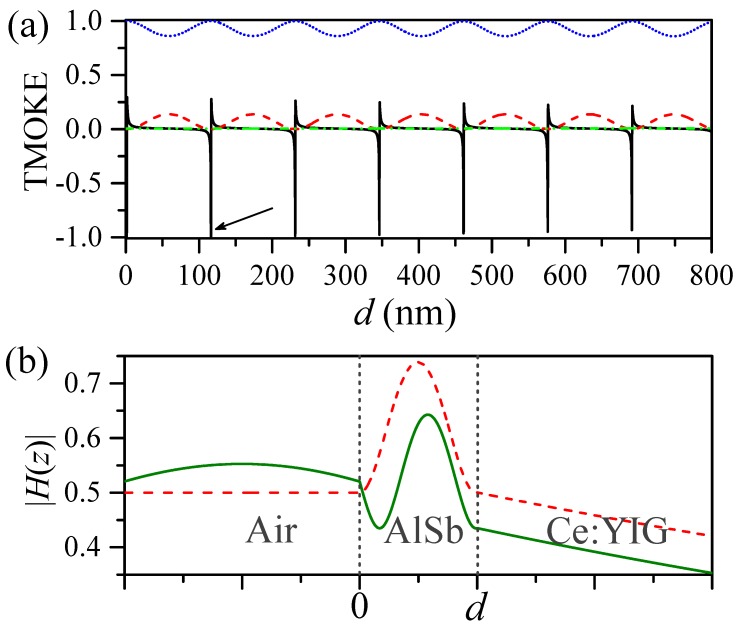
(color online) (**a**) The reflectance (dashed) and transmittance (dotted) spectra are shown, for m=−1, as a function of the AlSb film thickness. TMOKE spectrum as a function of the AlSb film thickness is represented by the solid (black) line. Nearly null absorbance (~$10^{-3}$) is numerically shown by the green dash-dot-dotted line in this figure. (**b**) Magnetic field distribution along the growth direction, *z*-axis, is shown for d=116.24 nm (dashed line) and d=140 nm (solid line). An enhancement of the electromagnetic field inside the high-refractive slab is observed at the resonance thickness (d=116.24 nm). Calculations were carried out by considering the incident medium as air with an incident angle $\theta =66.45^{\circ }$.

In Figure 3a we plot the TMOKE, reflectance, and transmittance spectra with solid (black), dashed (red), and dotted (blue) lines, respectively. Dash-dot-dotted line (green) presents numerical data for the absorbance, $A=1-T_{pp}-R_{pp}\approx 10^{-3}$, to evidence the extremely low level of losses in this structure. Results are calculated for $\theta =66.45^{\circ }$ and m=−1. Sharp Fano-like resonances in the TMOKE spectrum are observed around the transmittance peaks, in contrast to featureless and quite small TMOKE values far from Fabry–Perot resonances. Because the sign of γ in Equations (4) and (5) differs for opposite senses of the magnetization (see Equation (9)), the reflection dips and transmission peaks are shifted to smaller or higher film thicknesses with magnetic fields and the TMOKE signal becomes enhanced. We must remark here that γ also depends on the saturation magnetic field strength, which for the in-plane configuration is found below 300 Oe. The saturation field and magnetization ($M_{s}=150\pm 15$ emu cm${}^{-3}$) were measured at room temperature in [27]. The electromagnetic field enhancement, associated to Fabry–Perot resonances, can be noted from Figure 3b where, for comparison purposes, we plotted the magnetic field amplitude $\left|H_{z}\right|$ along the growth direction for a resonant d=116.2 nm (dashed line) and a non-resonant d=140 nm (solid line) HRI film thicknesses. The enhanced electromagnetic field, distributed inside the magnetic material, boosts MO activity, in analogy to the plasmonic approach. We must remark here the simplicity, and larger enhancements of the TMOKE signals, of this proposal when compared with two-dimensional dielectric MO gratings [33].

**Figure 4:**
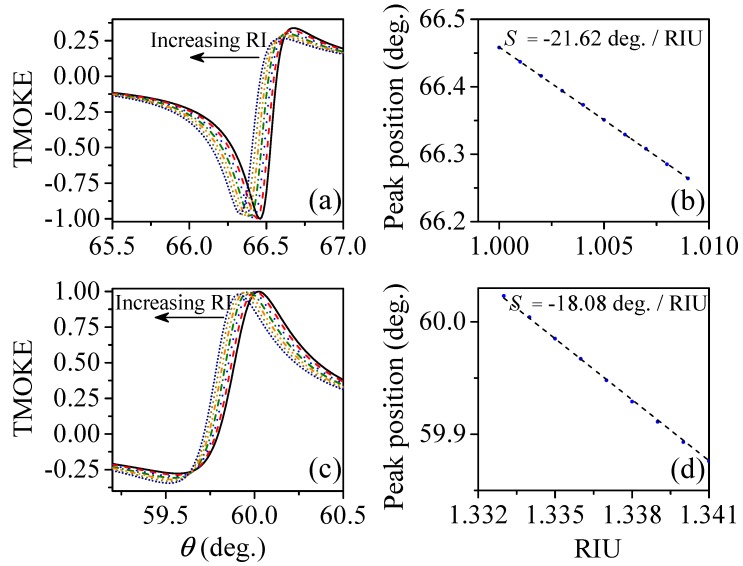
(color online) The system was first optimized to work in a gaseous incident medium, i.e., we considered the incident medium as air, with refractive index $n_{\mathrm{inc}}=1.0$. The AlSb film thickness was used as d=116.24 nm. Results are presented as: (**a**) TMOKE as a function of the angle of incidence for increasing refractive index of the incident medium in steps of $\Delta n_{\mathrm{inc}}=10^{-3}$. Successive peaks from the right to the left (indicated by the arrow) are for 1.000, 1.001, 1.002, 1.003, 1.004, 1.006 and 1.007. (**b**) Corresponding angular peak position and their linear fitting, with S=−21.62${}^{\circ }$/RIU. (**c**,**d**) show similar results but for the system optimized to work in an aqueous medium. For this latter case, calculations were performed using d=118.88 nm and $n_{\mathrm{inc}}=1.333$. $\Delta n_{\mathrm{inc}}$ was also considered as $10^{-3}$. The corresponding sensitivity was calculated as S=−18.08${}^{\circ }$/RIU.

To assess the suitability of our concept for sensing applications, we now consider small variations of the refractive index of the incident medium. For this latter purpose we considered two cases: (1) the system was optimized to work in air for gas sensors; (2) the system was optimized to work in an aqueous medium for detection of bioanalytes. In the first case, presented in Figure 4a, we show the shifts in the TMOKE peak due to small variations (step $\Delta n_{\mathrm{inc}}=10^{-3}$) of the refractive index of air ($n_{\mathrm{inc}}=1$), which are considered as due to the presence of low concentrations of a gaseous analyte. A linear variation of the TMOKE peak position, with increasing $n_{\mathrm{inc}}$, is shown in Figure 4b. The corresponding gas sensitivity, obtained from the slope of the linear fitting, is S=−21.62${}^{\circ }/$RIU (refractive index unit). For the second case, we considered $n_{\mathrm{inc}}=1.333$ varying also in steps of $\Delta n_{\mathrm{inc}}=10^{-3}$. Results for the TMOKE are presented in Figure 4c, while the corresponding peak position changes and sensitivity (S=−18.08${}^{\circ }/$RIU) are shown in Figure 4d. We expect that further studies inspired by this seminal work can reach improved detection limits, even higher than our previous magnetoplasmonic proposals [25,26] because of the lossless optical response.

## 2. Conclusions

To conclude, we theoretically demonstrated a novel dielectric structure for lossless giant enhancement of the transverse magneto-optical Kerr effect. It is based on magnetic dielectric substrate covered with a high-refractive index dielectric film. The high-refractive index contrast provides a set of Fabry–Perot resonances with extraordinary enhanced TMOKE values. The periodicity of Fabry–Perot resonances with the ratio between the cover film thickness (*d*) and the incident wavelength (λ) allows tunability and control of the magneto-optical response in the desired spectral region. This unique ability is advantageous for sensing and biosensing applications, as numerically shown in this work.
